# NKX2-1 Is Required in the Embryonic Septum for Cholinergic System Development, Learning, and Memory

**DOI:** 10.1016/j.celrep.2017.07.053

**Published:** 2017-08-15

**Authors:** Lorenza Magno, Caswell Barry, Christoph Schmidt-Hieber, Polyvios Theodotou, Michael Häusser, Nicoletta Kessaris

**Affiliations:** 1 Wolfson Institute for Biomedical Research, University College London, Gower Street, London WC1E 6BT, UK; 2 Department of Cell and Developmental Biology, University College London, Gower Street, London WC1E 6BT, UK; 3 Department of Neuroscience, Physiology and Pharmacology, University College London, Gower Street, London WC1E 6BT, UK

**Keywords:** septum, hippocampus, theta, acetylcholine, development

## Abstract

The transcription factor NKX2-1 is best known for its role in the specification of subsets of cortical, striatal, and pallidal neurons. We demonstrate through genetic fate mapping and intersectional focal septal deletion that NKX2-1 is selectively required in the embryonic septal neuroepithelium for the development of cholinergic septohippocampal projection neurons and large subsets of basal forebrain cholinergic neurons. In the absence of NKX2-1, these neurons fail to develop, causing alterations in hippocampal theta rhythms and severe deficiencies in learning and memory. Our results demonstrate that learning and memory are dependent on NKX2-1 function in the embryonic septum and suggest that cognitive deficiencies that are sometimes associated with pathogenic mutations in NKX2-1 in humans may be a direct consequence of loss of NKX2-1 function.

## Highlights

• Forebrain cholinergic neuron subsets originate from septal NKX2-1^+ve^ progenitors
• Septal *Nkx2-1* deletion causes widespread loss of forebrain cholinergic neurons
• Severe deficits in learning and memory in septal *Nkx2-1* conditional mutant mice
• Hippocampal network activity alterations in the absence of embryonic septal NKX2-1

## Graphical Abstract

**Figure:**
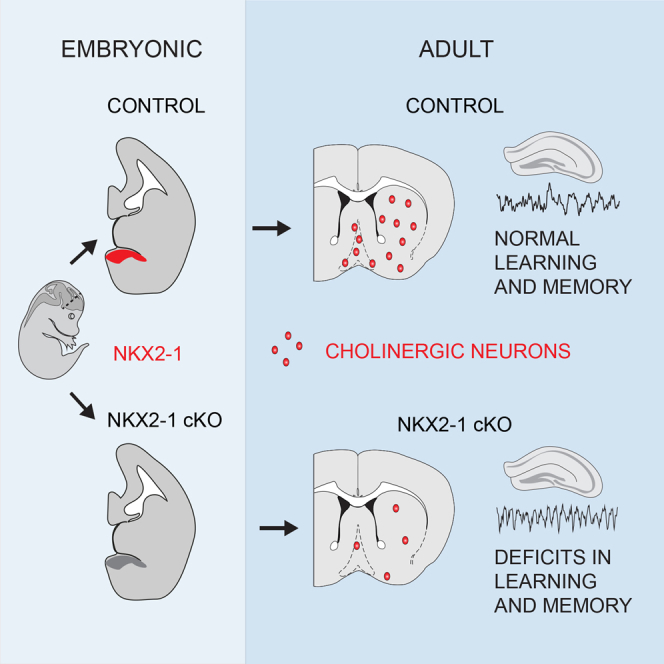

## Teaser

*NKX2-1* is a highly conserved patterning gene in the developing forebrain, mutations in which can lead to a spectrum of disorders including cognitive deficiencies. Using genetic fate mapping and intersectional deletion, Magno et al. demonstrate a requirement for embryonic septal NKX2-1 in forebrain cholinergic system development and learning and memory.

## Introduction

*Nkx2-1* (also known as *Ttf1*, *Titf-1*, or *Tebp*) is a widely conserved homeobox-encoding “hub gene” with a high degree of connectivity and functional significance during embryogenesis (Kang et al., 2011). Mutations in *NKX2-1* in humans account for over 50% of cases presented with the rare autosomal dominant movement disorder benign hereditary chorea (BHC) (Inzelberg et al., 2011, Kleiner-Fisman and Lang, 2007, Peall and Kurian, 2015). More recently, psychiatric symptoms, as well as cognitive deficiencies that include mental retardation (Gras et al., 2012), learning difficulties (Gras et al., 2012), and memory deficits (Sempere et al., 2013) have been identified in individuals with mutations in NKX2-1, raising the possibility that NKX2-1 may be required for development of the cognitive system.

NKX2-1 orchestrates the development of the medial ganglionic eminence (MGE)—one of the main sites of expression of this gene—by repressing alternative neuroepithelial identities and activating MGE-specific transcriptional programs (Butt et al., 2008, Kessaris et al., 2014, Sandberg et al., 2016, Sussel et al., 1999). In the absence of NKX2-1, the MGE becomes respecified into alternative lateral ganglionic eminence (LGE)-like fates and downstream MGE-specific genes, some of which are direct targets of NKX2-1, fail to be activated (Du et al., 2008, Sandberg et al., 2016, Sussel et al., 1999). Hence, NKX2-1 constitutes one of the main factors that pattern the ventral forebrain and parcellate its germinal zones into functionally distinct progenitor pools.

The NKX2-1 neuroepithelial zone has been subdivided into several subdomains based on the combinatorial expression of a number of transcription factors (Flames et al., 2007). Although there is little evidence to date for entirely distinct neuronal fates arising from each domain, there are clear biases in neuronal subtype generation: for example, the dorsal MGE generates many more somatostatin (SST)-expressing cortical interneurons than parvalbumin (PV)-expressing ones compared to the ventral MGE (Flames et al., 2007, Fogarty et al., 2007). Similarly, preoptic area (POA) progenitors expressing NKX2-1 generate neurons of the globus pallidus, but only few interneurons for the cortex (Flandin et al., 2010). NKX2-1 is also expressed in the septal neuroepithelium (Flames et al., 2007, Rubin et al., 2010), where its function and the identity of neurons derived from it remain unexplored.

The adult septal complex and, in particular, the medial septum and vertical limb of the diagonal band (MSvDB), which contain the septohippocampal projection system, constitute one of the major subcortical brain areas that regulate learning and memory (Brandner and Schenk, 1998). Septohippocampal projections orchestrate hippocampal physiology by modulating synaptic plasticity and transmission (Colom, 2006, Drever et al., 2011, Nicoll, 1985). At a network level, the MSvDB provides a rhythmic input that drives the synchronous firing of hippocampal neurons, producing a prominent oscillatory brain activity known as theta rhythm (Buzsáki, 2002, Colom, 2006, Yoder and Pang, 2005). Hippocampal theta can be detected during voluntary movement (Whishaw and Vanderwolf, 1973) or highly aroused states (Buzsáki, 2005) and has been associated with navigation, spatial learning, and memory processes in humans and other species (Buzsáki, 2005, Colgin, 2013, Hartley et al., 2013, Hasselmo, 2005, O’Keefe and Nadel, 1978). In accordance with this, septal lesions that suppress theta also lead to impairments in memory (Mitchell et al., 1982).

We know very little about the development of the septum during embryogenesis and the origin of its constituent neurons. In view of the pioneering roles of NKX2-1 in neuronal fate determination, the pivotal role of the septum in cognition, and the cognitive deficiencies reported in human patients with pathogenic NKX2-1 mutations, we sought to determine the role of NKX2-1 specifically within the septal neuroepithelium. We demonstrate through genetic fate mapping and conditional mutagenesis in mice that NKX2-1 is selectively required for the development of septal cholinergic, but not GABAergic or glutamatergic septohippocampal projection neurons. In addition, septal NKX2-1 neuroepithelial cells generate large cohorts of cholinergic neurons for the striatum and other forebrain cholinergic centers. In the absence of NKX2-1, these neurons fail to form and mice demonstrate alternations in hippocampal theta rhythms and deficits in learning and memory. Our data demonstrate that the septal NKX2-1-expressing neuroepithelium has a neurogenic potential that is partly distinct from other NKX2-1 progenitor domains. In addition, we provide evidence for a causal link between NKX2-1 deficiency and memory deficits and suggest that cognitive impairment may constitute an integral phenotype of BHC and the broader spectrum of NKX2-1-related disorders.

## Results

### Cholinergic, but Not GABAergic or Glutamatergic, MSvDB Neurons Originate from Septal *Nkx2-1*-Expressing Neuroepithelial Cells

**Figure 1:**
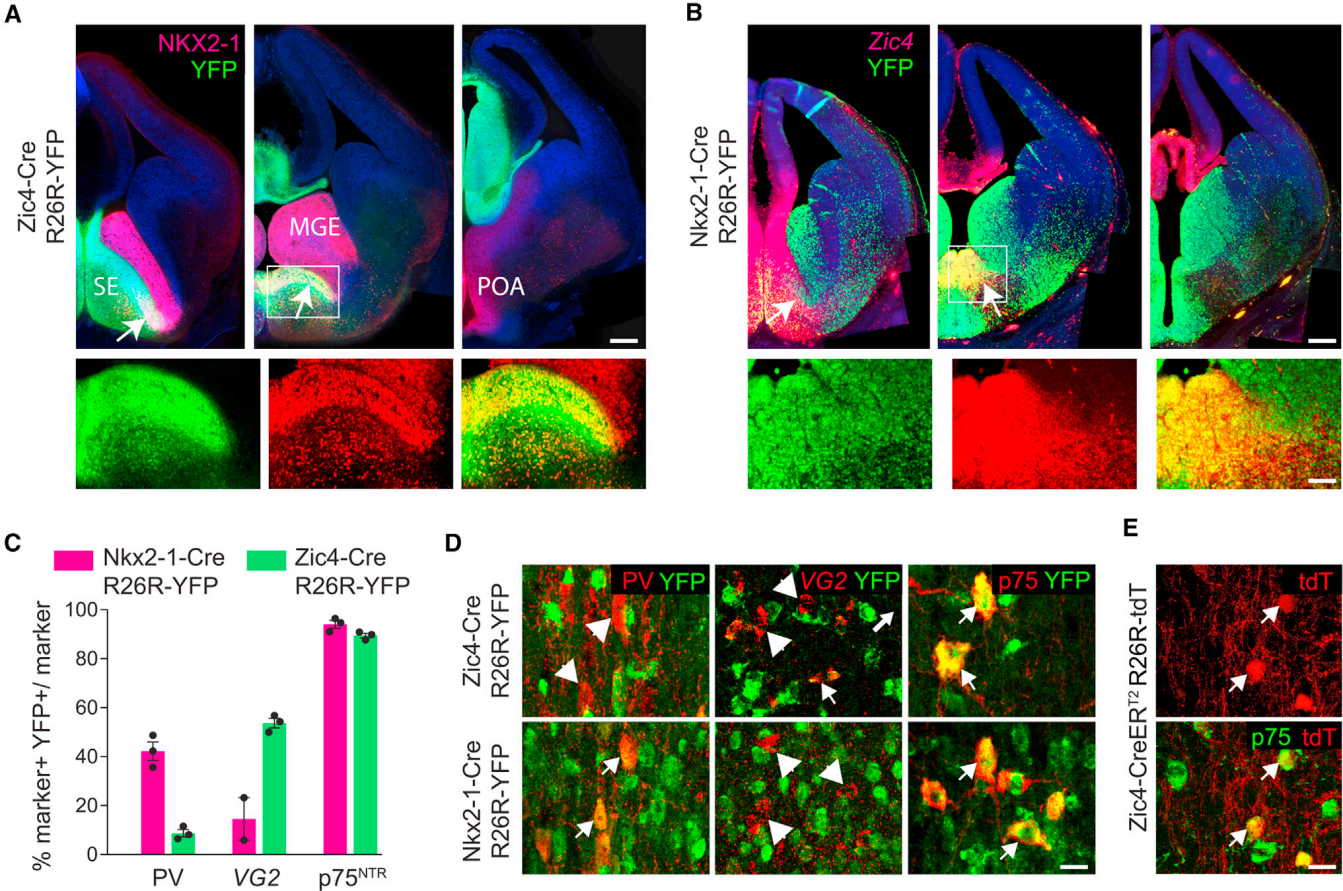
The Embryonic Origin of MSvDB Septohippocampal Projection Neurons (A) Expression of NKX2-1 in the MGE and in a caudo-ventral domain of the septal neuroepithelium (E13.5). The entire septum is labeled with YFP in Zic4-Cre;R26R-YFP mice. The overlap between NKX2-1 and YFP is restricted to the septum (arrows). The boxed areas in (A) and (B) are shown in higher magnification. (B) In Nkx2-1-Cre;R26R-YFP E13.5 embryos, expression of YFP is detected in the MGE, the POA, and the caudo-ventral septum that co-expresses *Zic4* (arrows). (C) Quantification of the contribution of Nkx2-1-Cre- and Zic4-Cre-expressing progenitors to MSvDB neurons expressing PV, *VG2*, and p75^NTR^. Data show mean ± SEM. (D) Double IHC for YFP and PV or p75^NTR^ and IHC for YFP followed by ISH for *VG2* in adult Zic4-Cre;R26R-YFP and Nkx2-1-Cre;R26R-YFP animals. (E) P75^NTR^ cholinergic neurons of the MSvDB co-express tdTomato (tdT) in Zic4-CreER^T2^;R26R-tdT mice where recombination had been induced at E9.5. Scale bars: 100 μm, (higher magnifications 50 μm) in (A) and (B) and 20 μm in (D) and (E). See also Figure S1.

Within the developing septum, expression of NKX2-1 can be detected in the subpallial septal neuroepithelium (Figure 1A). This region co-expresses the transcription factor ZIC4 and can be labeled with yellow fluorescent protein (YFP) in Zic4-Cre;R26R-YFP mice (Rubin et al., 2010) (Figure 1A). In order to identify septal neurons generated from *Nkx2-1*-expressing precursors, we crossed Nkx2-1-Cre mice (Kessaris et al., 2006) to the ROSA26 (R26R)-YFP reporter (Figure 1B). Given that expression of *Nkx2-1* is not restricted to the septum, but extends to the MGE and the POA, we compared this fate map to that of Zic4-Cre;R26R-YFP in order to identify cell types labeled in both mice and hence derived specifically from the septal NKX2-1 progenitor zone. We quantified the expression of YFP in the three major septohippocampal projection neurons of the MSvDB (Figure 1C). ∼40% of GABAergic PV-expressing neurons, which form the main projection to the hippocampus, co-expressed YFP in Nkx2-1-Cre mice (Figures 1C and 1D). However, fewer than 10% are labeled in Zic4-Cre;R26R-YFP mice, indicating that most of these cells are generated in *Nkx2-1*-expressing domains outside the septum (Figures 1C and 1D). A large number of VGluT2(*VG2*)-expressing glutamatergic neurons of the MSvDB were labeled in Zic4-Cre;R26R-YFP mice, indicating septal origin, but a very small number of those originate from *Nkx2-1* precursors (Figures 1C and 1D). In contrast, nearly all MSvDB cholinergic neurons expressing the neurotrophin receptor p75^NTR^ were labeled in both transgenic mouse lines (Figures 1C and 1D). This suggested an exclusive origin for these cells within the NKX2-1-expressing domain of the septal neuroepithelium.

Postmitotic cholinergic neurons of the septum are thought to express ZIC1 and NKX2-1 (Wei et al., 2012), leaving open the possibility that these cells originate outside the septum, upregulate ZIC4 postmitotically, and hence are labeled in Zic4-Cre mice. In order to temporally control the Cre activity, we generated Zic4-CreER^T2^;R26R-tdTomato transgenic mice and induced recombination at embryonic day (E)9.5 (Figures S1A and S1B). tdTomato-expressing cells were restricted to the septum at early stages and were absent from the ventricular/subventricular zones (VZ/SVZ) or mantle of the MGE (Figure S1B). At postnatal day (P)30, numerous p75^NTR^-labeled neurons co-expressed the tomato reporter, confirming that these originated from neuroepithelial cells of the septum that expressed Zic4-CreER^T2^ at embryonic stages (Figure 1E).

### Large Contribution of Septal *Nkx2-1*-Expressing Neuroepithelial Cells to Striatal and Basal Forebrain Cholinergic Neurons

**Figure 2:**
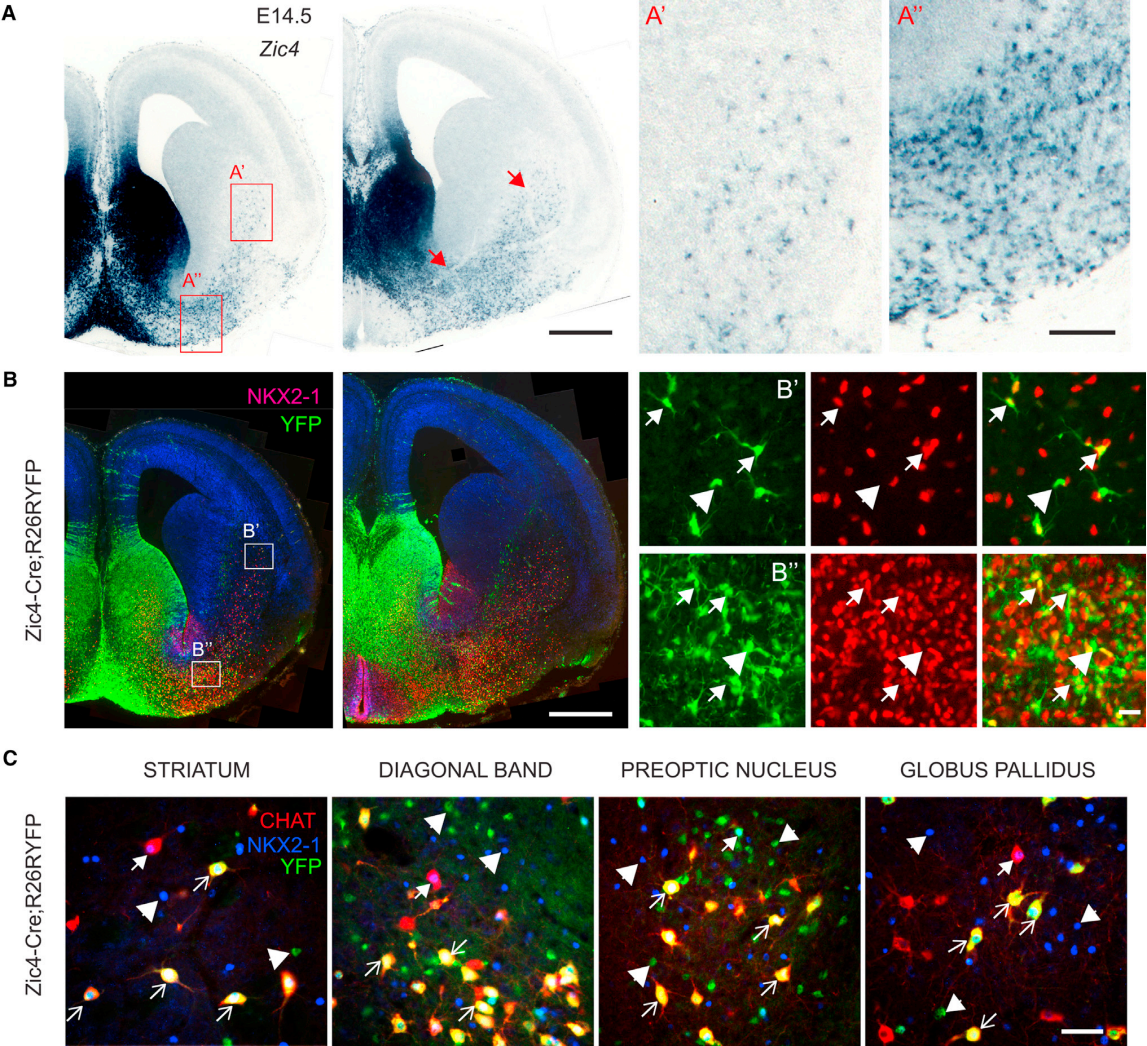
NKX2-1-Expressing Progenitors in the Septum Generate Cholinergic Neurons of the Basal Forebrain and Striatum (A) Expression of *Zic4* in the septum and in cells migrating toward the basal forebrain and striatum. (B) Septal-derived cells expressing YFP in Zic4-Cre;R26R-YFP embryos migrate to the striatum and basal forebrain and maintain expression of NKX2-1. (C) Triple IHC for CHAT, NKX2-1, and YFP in adult Zic4-Cre;R26R-YFP mice demonstrating septal contribution to different forebrain cholinergic centers. Scale bars: 500 μm in (A) and (B), 100 μm in (A”), 20 μm in (B”), and 50 μm in (C).

Previous work had shown that nearly all cholinergic projection neurons of the basal forebrain and interneurons of the striatum are generated from NKX2-1-expressing precursors (Fragkouli et al., 2009, Magno et al., 2009, Magno et al., 2011, Marin et al., 2000). To identify possible septal-derived cholinergic neurons outside the septum, we examined Zic4-Cre;R26R-YFP transgenic mice. At embryonic stages, we could detect *Zic4*- and YFP-expressing cells streaming away from the septum, seemingly migrating toward the striatum and basal forebrain (Figures 2A and 2B). Many YFP^+ve^ cells expressed NKX2-1, suggesting that they originated from the NKX2-1-expressing domain of the septal neuroepithelium (Figure 2B). At P30, numerous YFP^+ve^ NKX2-1^+ve^ neurons of the striatum, horizontal diagonal band, magnocellular preoptic nucleus, and globus pallidus/subtantia innominata in Zic4-Cre;R26R-YFP transgenic mice co-expressed choline acetyltransferase (CHAT), indicating their cholinergic identity (Figure 2C). These septal-derived CHAT^+ve^ neurons accounted for over 50% of all cholinergic neurons in each of these centers. Overall, the data demonstrate that the caudo-ventral domain of the septal neuroepithelium that expresses NKX2-1 is the source, not only of septo-hippocampal cholinergic projection neurons, but also of large subsets of striatal and other basal forebrain cholinergic neurons.

### Septal-Specific Ablation of *Nkx2-1*: Molecular Changes

**Figure 3:**
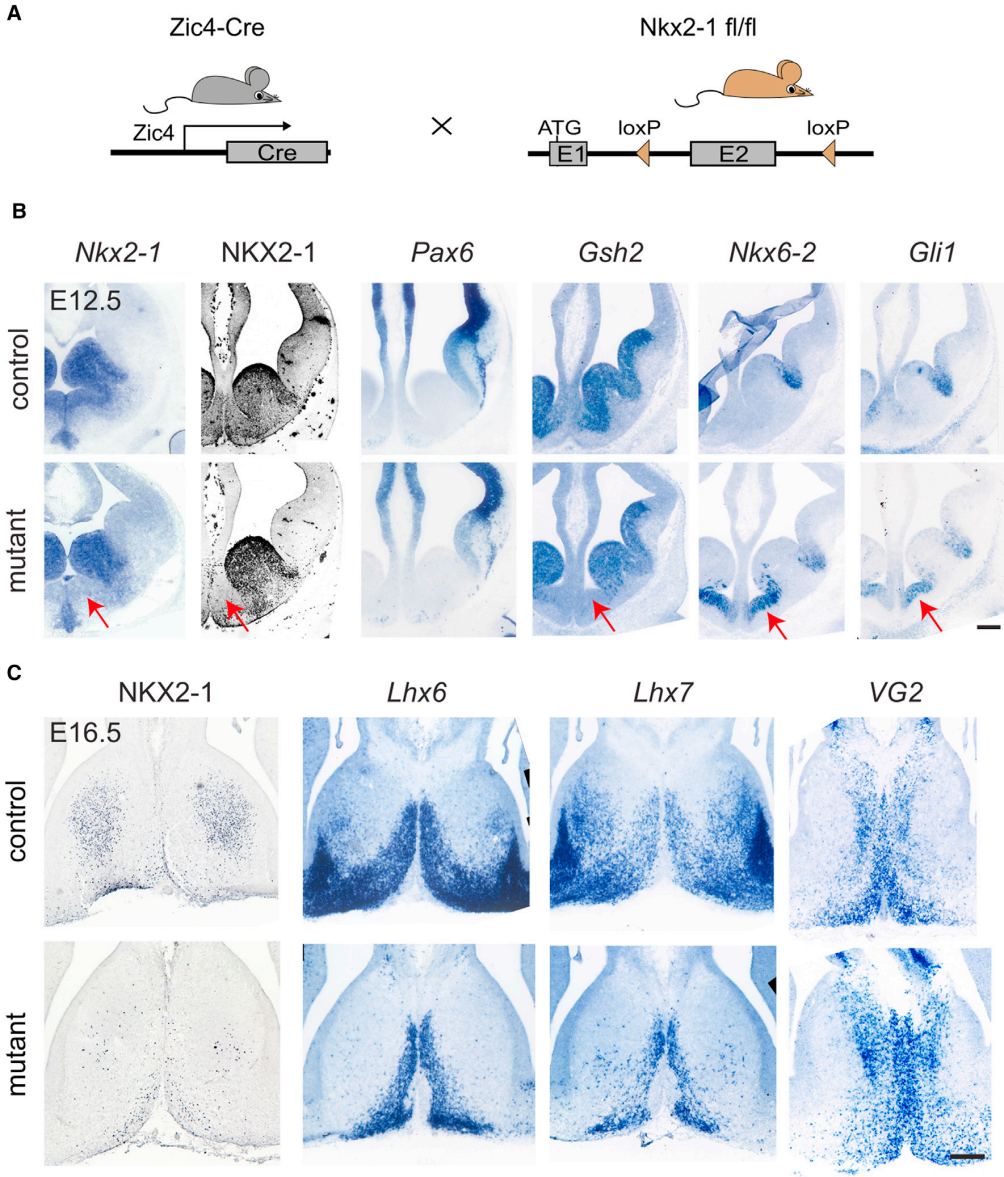
Conditional Deletion of *Nkx2-1* in the Developing Septum and Changes in Gene Expression (A) Breeding strategy for conditional deletion of *Nkx2-1* in septal precursors. (B) Neuroepithelial changes in gene expression in septal conditional mutant embryos (Zic4-Cre;Nkx2-1^fl/fl^) compared to controls (Zic4-Cre;Nkx2-1^fl/+^) at E12.5. ISH (blue signal), IHC (black signal). The loss of *Nkx2-1* mRNA and protein, reduction of *Gsh2*, and upregulation of *Nkx6-2* and *Gli1* (arrows) observed in the mutants are shown. (C) Gene expression changes in postmitotic neurons of the septum. ISH (blue), IHC (black). The loss of NKX2-1 and reduction of *Lhx6* and *Lhx7* expression observed in mutants are shown. Scale bars: 100 μm in (B) and 50 μm in (C).

In order to identify the function of NKX2-1 specifically within the septum, we bred Zic4-Cre mice with ones carrying a Cre-conditional loss-of-function (LOF) allele for *Nkx2-1* (Kusakabe et al., 2006) (Figure 3A). Resulting offspring showed restricted septal deletion of *Nkx2-1* and absence of NKX2-1 protein solely within the caudo-ventral domain of the developing septum (Figure 3B). We assessed expression of a range of markers that label septal neuroepithelial domains (Flames et al., 2007) in order to identify molecular changes caused by deletion of NKX2-1. Expression of the pallial marker *Pax6* remained unchanged, indicating the absence of major dorso-ventral patterning shifts (Figure 3B). However, a reduction of *Gsh2* expression and upregulation of *Nkx6-2* and *Gli1* were observed (Figure 3B), indicating a requirement for NKX2-1 in patterning of the septal neuroepithelium. In order to identify neuronal changes in conditional mutant mice, we assessed expression of postmitotic neuron markers of the septum. NKX2-1 itself, *Lhx6*,—a marker of bipotential GABAergic/cholinergic progenitors (Fragkouli et al., 2009)—and *Lhx7* were clearly lost in large populations of septal neurons, whereas expression of the glutamatergic marker *VG2* was unaffected (Figure 3C). Overall, our data demonstrate a requirement for NKX2-1 in genetic patterning of the septal neuroepithelium and specification of subsets of septal neurons.

### Septal NKX2-1 Is Selectively Required for Specification of Medial Septal and Large Subsets of Other Forebrain Cholinergic Neurons

**Figure 4:**
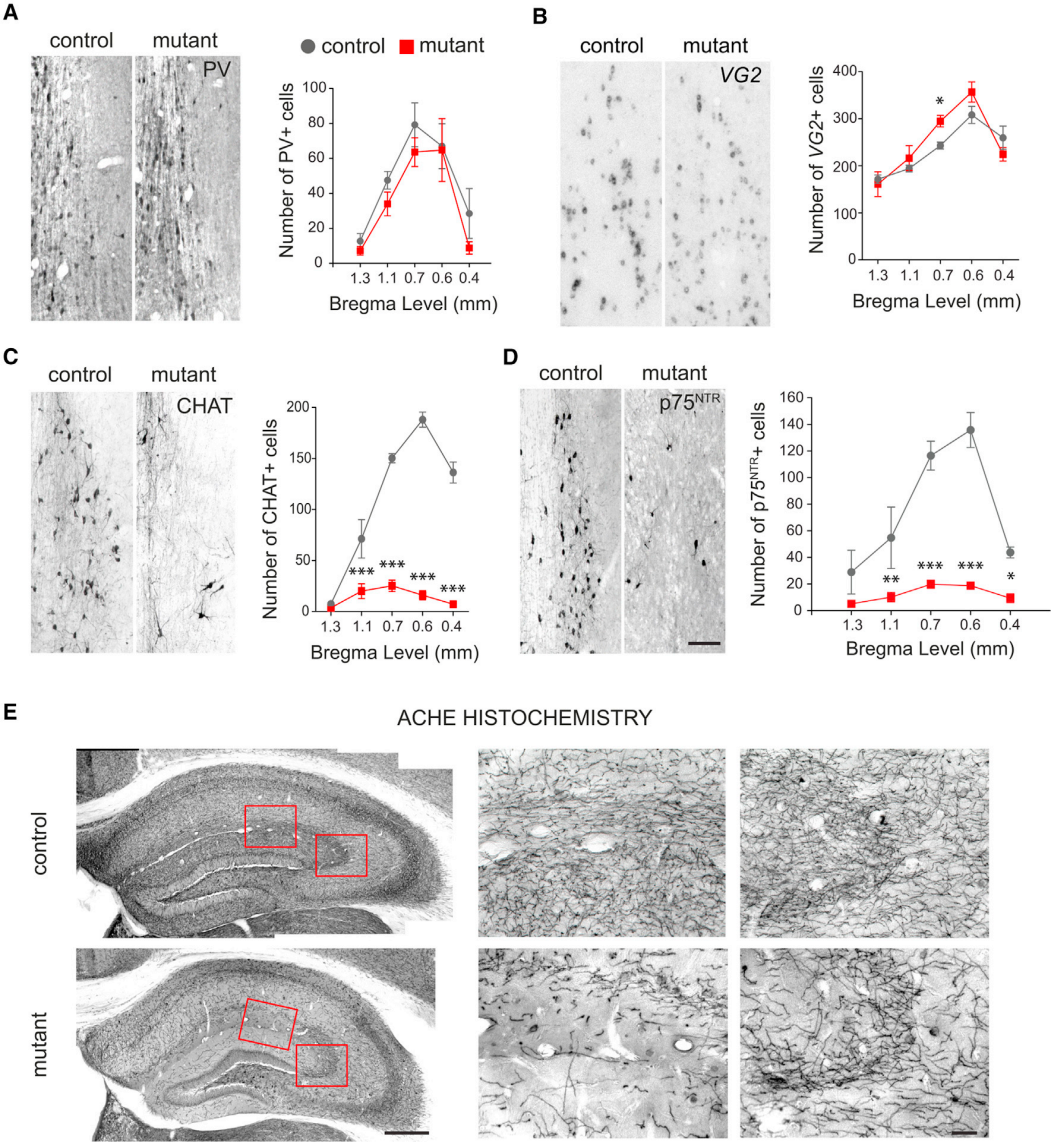
NKX2-1 Is Required for Development and Maintenance of Cholinergic, but Not GABAergic or Glutamatergic, Neurons of the MSvDB (A and B) Normal numbers of PV- (A) and *VG2*-expressing (B) neurons in the MSvDB of P30 mutant mice lacking NKX2-1 in the septum (control n = 4, mutant n = 5). Data show mean ± SEM. (C and D) Severe reduction of cholinergic CHAT- (C) and p75^NTR^-labeled (D) neurons at five septal rostro-caudal levels (mean ± SEM). Two-way RM ANOVA, post hoc uncorrected Fisher’s LSD, ^∗^p < 0.05, ^∗∗^p < 0.01, and ^∗∗∗^p < 0.0001. Scale bar: 50 μm. (E) AChE histochemistry at P90. The loss of cholinergic fibers in the hippocampus in mutant animals (boxed areas shown at higher magnification) is shown. Scale bars, 250 μm and 50 μm. See also Figure S2.

In order to identify and quantify mature neurons affected by loss of septal NKX2-1, we examined GABAergic, cholinergic, and glutamatergic neurons of the MSvDB. PV- (GABAergic) and *VG2* (glutamatergic)-expressing neurons were detected in comparable numbers in control and mutant mice (Figures 4A and 4B) (PV: two-way repeated measures [RM] ANOVA: genotype, F(1,7) = 1.48, p = 0.263; level, F(4,28) = 20.3, p < 0.0001; and interaction, F(4,28) = 0.354, p = 0.839. *VG2*: two-way RM ANOVA: genotype, F(1,7) = 0.921, p = 0.369; level, F(4,28) = 39.49, p < 0.0001; and interaction, F(4,28) = 3.074, p = 0.032). In contrast, CHAT^+ve^ cholinergic neurons were severely reduced in mutants compared to controls (Figure 4C) (two-way RM ANOVA: genotype, F(1,6) = 235.5, p < 0.0001; level, F(4,24) = 52.01, p < 0.0001; and interaction, F(4,24) = 40.82, p < 0.0001). Similarly, quantification using p75^NTR^ as an alternative marker for cholinergic neurons of the septum yielded the same results, confirming a consistent and selective loss of cholinergic markers in the septum (two-way RM ANOVA: genotype, F(1,7) = 32.69, p = 0.0007; level, F(4,28) = 4.65, p < 0.0001; and interaction, F(4,28) = 22.47, p < 0.0001) (Figure 4D). Consistent with the loss of septo-hippocampal cholinergic neurons, we detected cholinergic deafferentiation of hippocampal (Figure 4E) and parahippocampal areas (Figure S2A). In contrast, the numbers of non-cholinergic MSvDB neurons expressing calbindin (CB) or calretinin (CR) and targeting non-hippocampal regions were unchanged in mutants compared to controls (Figure S3A).

**Figure 5:**
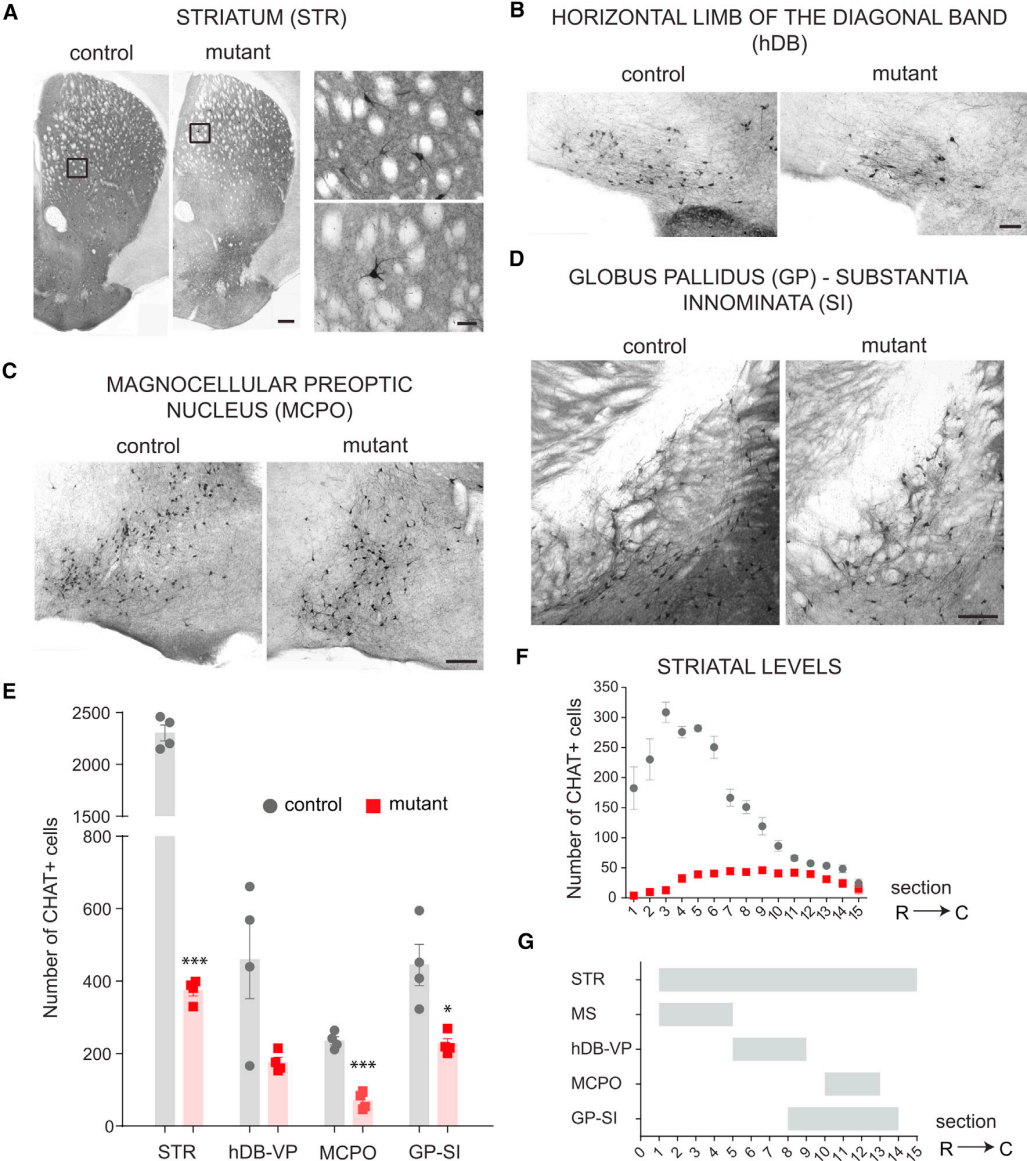
Loss of Septal NKX2-1 Causes Reduction of Forebrain Cholinergic Neurons (A–D) Reduction in CHAT^+ve^ neurons in controls and mutants at P90 in the rostral striatum (STR) (A), the horizontal diagonal band (hDB) (B), the preoptic nucleus (MCPO) (C), the globus pallidus (GP), and substantia innominata (SI) (D). (E) Quantification of CHAT^+ve^ neurons in the forebrain of controls and mutants at P90 (mean ± SEM; n = 4). Unpaired two-tailed t test: ^∗^p < 0.05 and ^∗∗∗^p < 0.0001. (F) Quantification of CHAT^+ve^ neurons throughout the rostro-caudal extent of the striatum (mean ± SEM). Two-way RM ANOVA. Post hoc uncorrected Fisher’s LSD, levels 1–9, ^∗∗∗^p < 0.0001; level 10, ^∗∗^p < 0.01; and levels 11–15, p > 0.05. (G) Gantt chart showing the levels of count for each region measured as the progressive numbering of sections from rostral to caudal levels. R-C, rostro-caudal. Scale bars: left 250 μm, right 50 μm in (A); 100 μm in (B); and 200 μm in (C) and (D).

Given the large contribution of the septal neuroepithelium to other cholinergic centers outside the septum, we quantified CHAT^+ve^ neurons in other forebrain areas. Reductions were detected in different regions, with rostral areas being more affected than caudal ones (Figure 5). These included the rostral striatum (Figures 5A, 5E, and 5F; two-way RM ANOVA: genotype, F(1,6) = 554.9, p < 0.0001; level, F(14,84) = 33.33, p < 0.0001; and interaction, F(14,84) = 36.62, p < 0.0001), the horizontal limb of the diagonal band and ventral pallidum (hDB-VP) (Figures 5B and 5E, p = 0.077), the preoptic nucleus (MCPO) (Figures 5C and 5E, p < 0.0001), and the globus pallidus-substantia innominata (GP-SI) (Figures 5D and 5E, p = 0.027). In contrast, “prototypic” GABAergic neurons of the globus pallidus, most of which express PV and originate from *Nkx2-1*-expressing precursors (Dodson et al., 2015), and MGE-derived GABAergic interneurons of the cortex, which express PV and SST (Fogarty et al., 2007) were unaffected (Figures S3B –S3D). This is consistent with the septally restricted ablation of *Nkx2-1* and the embryonic origin of these neurons outside the septum. Largely preserved acetylcholinesterase (AChE) staining was observed in the caudal striatum, amygdala, and cerebral cortex (Figure S2B). Overall, the data demonstrate that cholinergic neurons of the septum and large numbers of striatal and basal forebrain cholinergic populations originate in the septal neuroepithelium and require NKX2-1 for their normal development.

### Cognitive Impairments in Mice Lacking Septal NKX2-1

**Figure 6:**
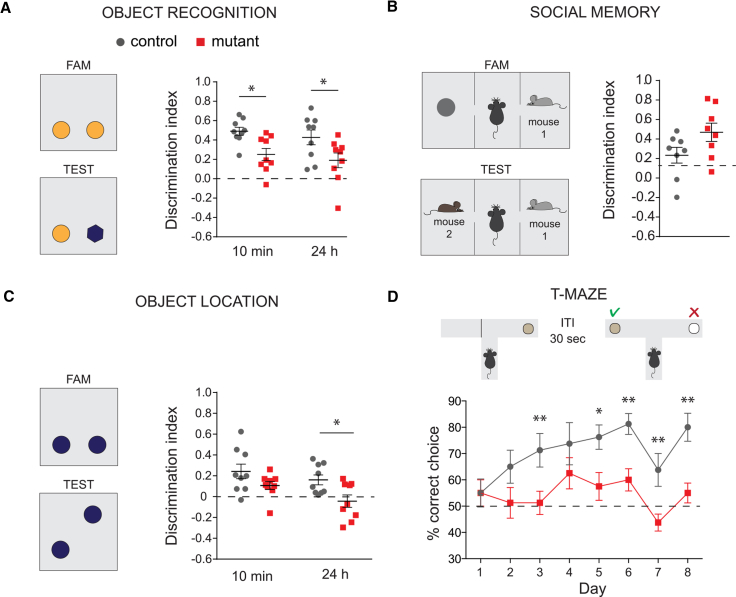
Embryonic Septal NKX2-1 Is Essential for Learning and Memory (A) NOR task with 10 min and 24 hr inter-trial intervals (n = 9). FAM, familiarization session, TEST, test session. Mutant mice show a lower discrimination index at both intervals. Mean discrimination index ± SEM. Two-way RM ANOVA. Post hoc uncorrected Fisher’s LSD, ^∗^p < 0.05, ^∗∗^p < 0.001, and ^∗∗∗^p < 0.0001. (B) Crawley’s social memory test (n = 8). The mutant mice display normal social memory. Mean discrimination index ± SEM, unpaired two-tailed t test. (C) Object location task with 10 min and 24 hr ITIs (n = 9). The mutant mice display lower discrimination index. Mean discrimination index ± SEM. Two-way RM ANOVA. Post hoc uncorrected Fisher’s LSD, ^∗^p < 0.05, ^∗∗^p < 0.001, and ^∗∗∗^p < 0.0001. (D) T-maze task over 8 consecutive days (n = 8). The mutant mice never perform above chance level (dashed line). The data show mean percentages of correct choices over each day ±SEM (ten trials per day). Two-way RM ANOVA. Post hoc uncorrected Fisher’s LSD, ^∗^p < 0.05, ^∗∗^p < 0.001, and ^∗∗∗^p < 0.0001. See also Figure S4 and Tables S1 and S2.

In view of the substantial loss of forebrain cholinergic neurons in mice lacking embryonic septal NKX2-1, and the contribution of the cholinergic system to learning and memory, we assessed control and mutant mice in a range of cognitive tests. Tests for locomotion and gross motor functions showed normal behavior in mutant mice (Figure S4). Although a general tendency for hyperactivity was detected in mutant mice in the first exposure to an open field, this recovered after subsequent exposures and increased habituation to the environment (Figure S4). The initial hyperactivity is consistent with recent findings suggesting a role for forebrain cholinergic neurons and a balanced cholinergic activity in the regulation of dopamine signaling and exploratory motor behavior (Patel et al., 2012). In a novel object recognition task (NOR) (with 10 min and 24 hr inter-trial intervals [ITIs] to assess short- and long-term memory), mutant mice showed a significantly lower discrimination index compared to controls at both ITIs (Figure 6A, two-way RM ANOVA: genotype, F(1,16) = 9.341, p = 0.007. Mutants versus controls, post hoc uncorrected Fisher’s least significant difference [LSD], 10 min ITI, p = 0.005; 24 hr ITI, p = 0.043). This was not caused either by changes in the predisposition to explore objects (no significant differences between mutants and controls in the mean exploration time during familiarization or test sessions; Table S1) or spontaneous preference for an object (assessed during the familiarization session; Table S2). In contrast, no difference was observed between mutants and controls when social memory was assessed in the three-chamber Crawley’s test, as both groups spent more time interacting with the novel subject over the familiar one (Figure 6B, one sample t test against no preference, controls, p = 0.023; mutants, p = 0.002; and controls versus mutants unpaired t test, p = 0.077).

To examine whether hippocampal-dependent spatial memory is affected, we tested mutant and control mice on a modified spatial version of the NOR task. The object location task (OLT) was carried out with two ITIs: 10 min and 24 hr (Figure 6C). Mutant mice showed a lower discrimination index compared to controls at 24 hr (Figure 6C, two-way RM ANOVA: genotype, F(1,16) = 10.86, p = 0.005; interval, F(1,16) = 3.897, p = 0.066; and interaction, F(1,16) = 0.352, p = 0.561. Mutants versus controls, post hoc uncorrected Fisher’s LSD, 10 min ITI, p = 0.093; and 24 hr ITI, p = 0.013). No significant differences between mutants and controls were detected in the mean exploration time (Table S1) or spontaneous preference for a position (Table S2).

Spatial memory was additionally assessed on a delayed non-matched to position memory task (Figure 6D, T-maze). In contrast to controls, mutant animals failed to exceed chance level performance, indicating a severe impairment in learning and spatial working memory (Figure 6D, one sample t test, data averaged across days versus theoretical mean, mutants, p = 0.065 and controls, p = 0.0003). Further, control mice exhibited a progressive improvement over the 8 days, and this was not shown by mutants (two-way RM ANOVA: genotype, F(1,14) = 19.17, p = 0.0006; day, F(7,98) = 3.37, p = 0.003; interaction, F(7,98) = 1.23, p = 0.294, Pearson’s r for mutants R^2^ = 0.006, p = 0.424; and Pearson’s r for controls R^2^ = 0.426, p = 0.039). Both mutants and controls showed a decreased number of correct responses during the T-maze test on day 7, which is suggestive of extraneous factors (e.g., excessive noise) (Figure 6D).

Taken together, our data indicate that deletion of *Nkx2-1* in the septum and consequent loss of cholinergic neurons in the MSvDB and other ventral forebrain regions cause severe impairment of learning and spatial memory, while social recognition and social memory are unaffected. These results point to a crucial role for embryonic septal NKX2-1 in hippocampal-dependent information processing and memory.

### Aberrant Hippocampal Theta Activity, but Normal Response to Novelty, in Freely Moving Mice Lacking Septal NKX2-1

**Figure 7:**
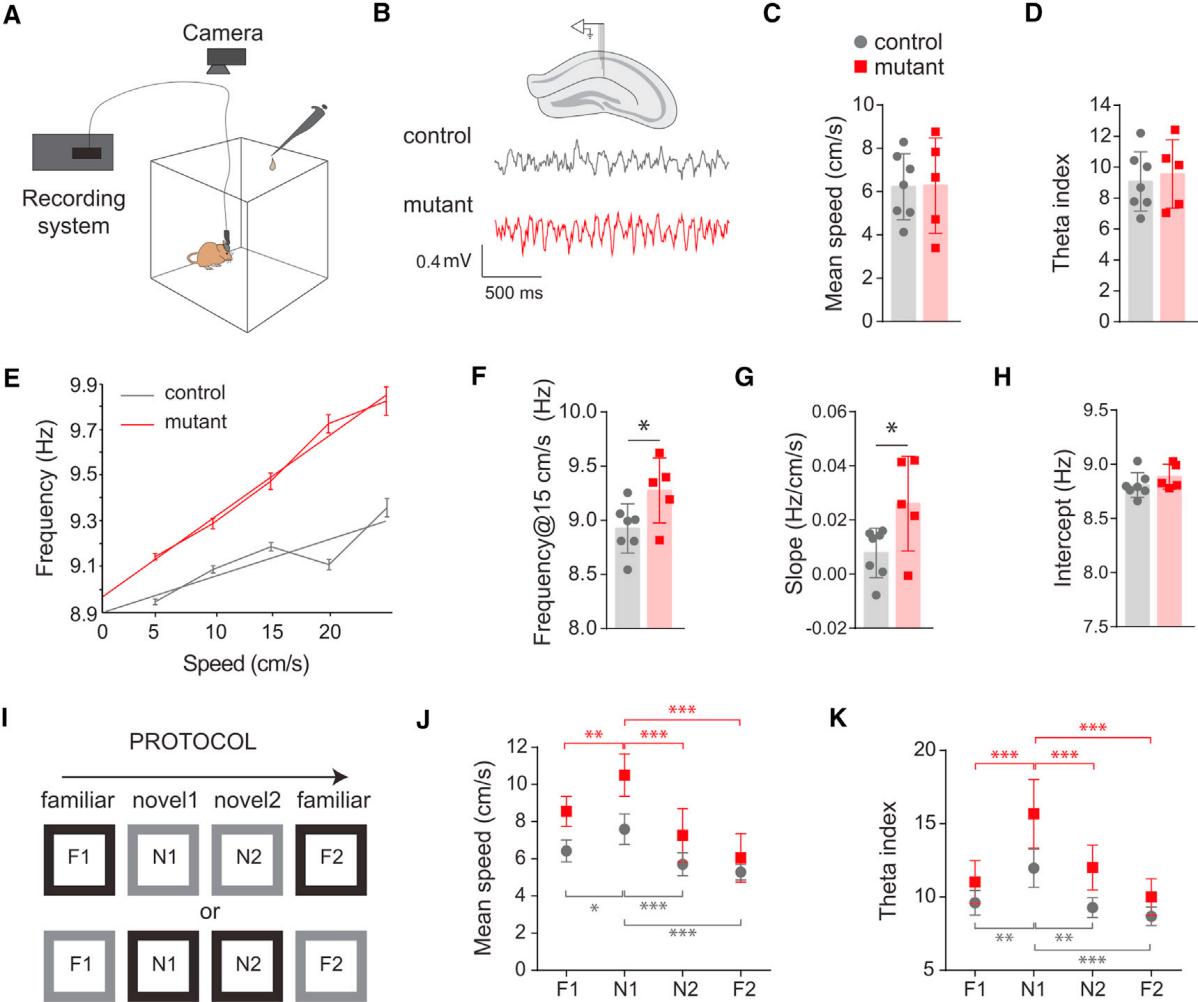
Altered Hippocampal Theta Activity, but Normal Response to Novelty, upon Septal Deletion of NKX2-1 (A) Apparatus for *in vivo* recordings (control n = 7, mutant n = 5). (B) Control and mutant mice show similar running speeds (average over four trials ±SEM). (C) Recording location in CA1 and raw traces of theta recording for a control and a mutant mouse. (D) No differences in theta index between mutants and controls (average over four trials ±SEM). (E) Frequency-speed function for a control and a mutant mouse showing the binned frequency data and linear fit. The mutant mice show higher frequency compared to controls at all speeds analyzed. (F) Theta frequency at 15 cm/s is increased in freely moving mutant mice. Unpaired two-tailed t test: ^∗^p < 0.05 (average over four trials ±SEM). (G) Mean slope of the frequency-speed function is increased in mutant mice. Unpaired two-tailed t test: ^∗^p < 0.05 (average over four trials ±SEM). (H) No difference in the intercept over the four trials (±SEM). (I) Experimental design for novelty detection. (J) Mutants and controls show comparable behavioral responses to novelty as detected by increased running speed during the first session in a novel environment. Mean speed ± SEM. Two-way RM ANOVA. Post hoc uncorrected Fisher’s LSD, ^∗^p < 0.05, ^∗∗^p < 0.001, and ^∗∗∗^p < 0.0001. (K) Theta index increases substantially during the first novel session for both mutants and controls (mean ± SEM). Two-way RM ANOVA. Post hoc uncorrected Fisher’s LSD, ^∗^p < 0.05, ^∗∗^p < 0.001, and ^∗∗∗^p < 0.0001.

Given the substantial loss of MSvDB cholinergic neurons, which constitute one of the three major septal projection components that modulate hippocampal theta rhythms, we examined the consequences of our genetic disruption on hippocampal network activity in freely moving mice in an open field arena (Figures 7A and 7B). Control and mutant mice traveled similar distances (data not shown) at comparable running speeds (Figure 7C, unpaired t test p = 0.962) and spent the same proportion of time moving within the arena (running speed >1 cm/s, controls, 84.3 ± 2.11%; mutants, 82.8 ± 5.12%; and unpaired t test p = 0.773) indicating normal motor behavior.

We recorded local field potentials (LFPs) *in vivo* from the hippocampal CA1 region of control and mutant mice (Figure 7B). Theta activity, measured as the theta index (the ratio of power in a 2 Hz band focused on the theta peak over power in the remainder of the 2 to 50 Hz range, see Experimental Procedures), was comparable between control and mutant mice over four trials (Figure 7D, unpaired t test p = 0.688). In contrast, theta frequency was higher in mutant mice compared to controls (Figure 7E). Theta frequency has been shown to be modulated by running speed and differences in frequency may result from systematic changes in running speed. To assess this possibility, we used linear regression to fit the running speed, theta frequency relationship for each animal (Figure 7F). Based on this fit, we extracted the theta frequency corresponding to a speed of 15 cm/s and used this as a basis for comparisons. We found that indeed the increase in theta frequency in the mutants is consistent and thus independent from any difference in the running speed (Figure 7F, unpaired t test p = 0.043). We also found a steeper modulation of frequency by speed in mutants compared to controls, as measured by the slope relating theta frequency to running speed (Figure 7G, unpaired t test p = 0.039). Finally, recent data have suggested that the intercept derived from the regression of theta frequency versus running speed can vary independently from the slope and reflects the anxiety level of the animal (Wells et al., 2013). However, we found no difference between mutants and controls in the intercept over the four trials (Figure 7H, unpaired t test p = 0.277). Taken together, these results indicate that reduction of cholinergic neurons of the septum and other forebrain regions caused by septal NKX2-1 deletion results in a shift of theta oscillation toward higher frequencies and a stronger modulation of theta by running speed.

Finally, as acetylcholine (ACh) is thought to be involved in novelty detection (Barry et al., 2012), we assessed theta dynamics in novel environments in our mutant mice. Control and mutant mice were trained to run in an open field box as described above, and were habituated to either a black box with a smooth floor or a gray box with a rough floor (counterbalanced between genotypes). For the first novel session, the boxes were swapped (Figure 7I). Both mutants and controls showed enhanced exploratory behavior (i.e., increased speed) during the first exposure to the novel environment (Figure 7J, two-way RM ANOVA: genotype, F(1,10) = 2.534, p = 0.143; trial, F(3,30) = 30.56, p < 0.0001; and interaction, F(3,30) = 2.953 p = 0.048). In addition, we detected significant changes in theta index in both controls and mutants in novel versus familiar trials, indicating clear changes at network level elicited by novelty (Figure 7K two-way RM ANOVA: genotype, F(1,10) = 2.117, p = 0.176; trial, F(3,30) = 1.404, p < 0.0001; and interaction, F(3,30) = 1.151, p = 0.232). These changes are in accordance with a transient increase of theta activity in novel environments and a decline of this effect in parallel with a reduction of the initial novelty. In summary, the data show that despite the loss of septal cholinergic neurons caused by deletion of NKX2-1 in the septum, mutant mice display normal responses to novelty.

## Discussion

We demonstrate that nearly all cholinergic projection neurons of the MSvDB and large subsets of other forebrain cholinergic neurons are derived from the septal neuroepithelial domain that expresses NKX2-1. In contrast, GABAergic and glutamatergic septohippocampal projection neurons are generated outside this region. Conditional deletion of *Nkx2-1* in the septum results in extensive depletion of its cholinergic progeny, alterations in hippocampal theta rhythm, and severe deficits in learning and memory. This demonstrates that NKX2-1 is essential in the embryonic septal neuroepithelium for the development of forebrain cholinergic neurons and their contribution to learning and memory.

NKX2-1 has been widely known as a key patterning factor in the forebrain neuroepithelium (Sussel et al., 1999). Within the VZ, NKX2-1 promotes regional identity by repressing genes that encode transcription factors and regulators of major signaling pathways, such as SHH, WNT, and BMP (Sandberg et al., 2016). It also promotes VZ/SVZ cell fates by activating MGE-specific genes (Sandberg et al., 2016). Consistent with these observations, we found upregulation of the SHH-activated genes and downregulation of downstream targets in NKX2-1-depleted zones (Du et al., 2008, Sandberg et al., 2016). This indicates that NKX2-1 in the septum and MGE promotes cell fates by regulating common downstream genes. The molecular mechanisms that regulate distinct cell fates downstream of NKX2-1 remain unknown. Identification of cell-type-specific co-factors and targets will elucidate such programs that drive distinct neuronal subtype identities.

Recent fate mapping work had suggested that GABAergic PV-expressing septal neurons originate in the MGE and POA (Wei et al., 2012), and our fate mapping is in agreement with an extra-septal origin of PV MSvDB neurons. However, the same study had suggested that cholinergic neurons of the septum, all of which express ZIC1 and NKX2-1, originate in the MGE and migrate tangentially to reach the septum. Several lines of evidence presented here indicate a septal NKX2-1-expressing origin of MSvDB cholinergic neurons: (1) nearly all are labeled in both Nkx2-1-Cre;R26R-YFP and the septal-specific Zic4-Cre;R26R-YFP mice, (2) early induction of Cre reporter expression within the septal neuroepithelium prior to cholinergic neuron birth resulted in labeling of these neurons, and (3) absence of *Zic4* expression in or near the embryonic VZ/SVZ of the MGE, suggesting that *Zic4* is not upregulated in postmitotic MGE derivatives. In fact, *Zic4*-expressing cells were observed migrating out of the septum toward the basal ganglia and basal forebrain. The reason for the discrepancy between the two studies is unknown. It remains possible that even though the transgenic mouse used by Wei et al. (2012) did not appear to express Cre within the entire septal NKX2-1 domain of the neuroepithelium—one of the main arguments for the proposed extra-septal origin—its postmitotic derivatives did, resulting in complete recombination of MS cholinergic neurons.

Forebrain cholinergic neurons originate from NKX2-1-expressing progenitors, but the contribution of neuroepithelial subdomains to different centers is unknown. Remaining cholinergic neurons in our conditional mutants lacking NKX2-1 in the septum must derive from the MGE and/or the POA, thereby suggesting heterogeneity in their origin. Forebrain cholinergic neurons comprise interneurons and projection neurons, however, their septal versus extra-septal origin cannot be the driver of this diversity since we found a mixed neuroepithelial origin for both interneurons (e.g., in the striatum), as well as projection neurons (e.g., in the hDB or substantia innominata).

Our findings appear to challenge the conclusions of previous studies showing a reduction in the occurrence and amplitude of theta *in vivo* following cholinergic ablation in the septum (Bassant et al., 1995, Lee et al., 1994) and the correlation of these changes with encoding, novelty detection, and memory performance (Düzel et al., 2010). However, these studies relied on the use of saporin-immunotoxic lesions and pharmacological interventions, manipulations that may have confounded the cholinergic effects through non-specific influences on glutamatergic and/or GABAergic neurons of the septum. Although the genetic approach applied in our study overcomes such limitations, the effect is nevertheless developmental and not temporally specific. As such, homeostatic mechanisms may allow for some recovery of cholinergic function and theta power albeit insufficient for normal execution of hippocampal-dependent spatial memory tasks.

Our data demonstrate that NKX2-1 is essential for spatial learning and memory through its requirement for specification of septal and extra-septal cholinergic neurons during embryogenesis. This is in accordance with several studies demonstrating an essential role for the basal forebrain cholinergic system in memory (Deiana et al., 2011, Hasselmo and Sarter, 2011). The severe deficiency in forebrain cholinergic neurons in our conditional mutant mice may impact on learning and memory directly by affecting plasticity (Hasselmo, 2006). Moreover, the observed shift in the frequency of theta oscillations and the stronger modulation by running speed may cause impairment in hippocampal processing of spatial information and the formation of new memories in mutant animals (Hartley et al., 2013). Although the changes in ACh can alone produce the observed deficiencies in learning and memory, other as yet unidentified alterations or homeostatic changes in the system may also contribute to the phenotype.

In sum, we provide evidence for a function for septal NKX2-1 in the development of the cognitive system. In addition, our findings raise the possibility that memory loss and learning impairments observed in patients with pathogenic NKX2-1 mutations may be a direct consequence of aberrant NKX2-1 function in the embryo and a resulting loss of forebrain cholinergic neurons.

## Experimental Procedures

### Animals

Zic4-Cre (Rubin et al., 2010), Nkx2-1-Cre (Kessaris et al., 2006), Nkx2-1 conditional mutant mice (Kusakabe et al., 2006), R26R-YFP (The Jackson Laboratory JAX 006148) (Srinivas et al., 2001), and R26R-tdTomato reporter mice (JAX 007914) (Madisen et al., 2010) have been described previously. Zic4-CreER^T2^ bacterial artificial chromosome (BAC) transgenic mice were generated as described previously (Rubin et al., 2010). Animals were maintained on a mixed C57BL6/CBA background at the Wolfson Institute for Biomedical Research in accordance with United Kingdom legislation (ASPA 1986). Male adult mice (older than 2 months) were used for behavioral and electrophysiological tests. Male and female mice were used in all other experiments.

### Tissue Processing and Immunohistochemistry

Tissue processing, immunohistochemistry (IHC), and *in situ* hybridization (ISH) were carried out as previously described (Magno et al., 2012, Rubin et al., 2010). Primary antibodies used were the following: rat anti-GFP IgG2a (1:1,000 cat # 04404-26; Nacalai Tesque, Kyoto, Japan), mouse anti-calbindin, rabbit anti-calretinin, and mouse anti-PV (all 1:1,000 cat # 300, 7697, 235 Swant, Bellizona, Switzerland), rabbit anti-p75^NTR^ (1:1,000 cat # G3231 Promega, Southampton, UK), goat anti-CHAT (1:100 cat # AB144P Merck Millipore, Darmstadt, Germany), rabbit anti-TTF-1 (1:100 cat # sc-13040 Insight Biotechnology, Wembley, UK). Alexa Fluor conjugated secondary antibodies used at 1:1,000 (Invitrogen, Carlsbad, CA, USA). For RNA, ISH digoxigenin (DIG)-labeled probes were used to detect *Nkx2-1* (probe spanned exon 2 which is deleted following Cre-mediated recombination), *Zic4*, *Pax6*, *Gsh2*, *Nkx6-2*, *Gli1*, *Lhx6*, *Lhx7*, *VG2*, and *Sst* transcripts. AChE histochemistry was carried out as described (Magno et al., 2011). Cell quantification was performed as described (Magno et al., 2012). See also Supplemental Information.

### Induction of CreER^T2^ by Tamoxifen

Tamoxifen was dissolved in corn oil by sonication for 30 min. A single dose of 320 mg/Kg was administered to pregnant females by oral gavage for analysis at embryonic stages. For analysis at postnatal stages, pregnant females received a single dose of 240 mg/Kg by oral gavage, the pups were delivered by caesarean section at E18.5, and reared by a foster mother.

### *In Vivo* Electrophysiological Recordings

The surgical procedure was performed on 10- to 15-week-old mice as previously described (Barry et al., 2007). Mice were chronically implanted with a single microdrive carrying four tetrodes constructed from 25 μm HM-L-coated platinum-iridium wire (90%/10%, California Fine Wire, Grover Beach, CA, USA) and electroplated in a platinum solution to <150 kΩ impedance. The implant was aimed at CA1 with the tips staggered to span a dorso-ventral extent from the stratum oriens to the hippocampal fissure. Coordinates for the CA1 target insertion zone were: 1.9 mm posterior to bregma, 1.5 mm lateral to the midline with a depth of 1.5 mm (for the deepest tetrode). Electrophysiological and positional data were acquired using an Axona recording system (Axona, St. Albans, UK), details of which have been described (Barry et al., 2012). Recording sites were confirmed by postmortem histological analysis.

Before the period of formal recording of the test trial series, mice were acclimatized to the recording apparatus (subsequently used as familiar environment for the novelty tests) and food restricted. Mice were pretrained to forage for drops of condensed milk/water 50:50. Each mouse was given multiple trials (5–10 min long) of training over the course of several days until it was fluent at the random foraging task, moving freely across all portions of the environment. All recordings took place in a dimly lit room within one of two open field arenas: a black wooden box with a smooth floor or a gray wooden box with a rough floor (both 40 × 40 × 40 cm). Testing was performed over 2 consecutive days after 2 days of acclimatization. On day 1, the animals were exposed to the familiar environment for four 10 min trials. On day 2, mice again received four 10 min trials, the first and fourth being in the familiar environment, with the second and third trial in a novel environment (Figure 4I, counterbalanced across animals). In between trials, mice were placed in a plastic holding box with sawdust bedding.

Analysis of the LFPs was carried out as previously described (Jeewajee et al., 2008). See also Supplemental Information. Theta frequency was taken as the frequency with peak power in a broad band including movement-related theta (7–11 Hz). All analyses were conducted using MATLAB 2015a (The MathWorks, Cambridge, UK). A theta index was calculated as the ratio of power in a 2 Hz band focused on the theta peak versus power in the remainder of the 2 to 50 Hz range or 2 to 125 Hz. Since the results obtained with the two methods provided similar results, only data for the 2–50 Hz theta index are shown.

### Behavioral Analysis

All animals used were broadly assessed for general health, sensory, and motor function at 3 months and no differences were observed between the genotypes. Experiments took place between 09:00–17:00 in a room where external sounds were masked by white noise. Sessions were video recorded for analysis.

#### Open Field

Analysis was carried out in an acrylic 30 × 30 × 40 cm square transparent box over 2 consecutive days (30 min on the first day and 10 min on the second day). ActualTrack software (Actual Analytics, Edinburgh, UK) was used to track mouse movements. The average speed was calculated as total distance traveled over the time spent moving.

#### OLT and NOR

Following habituation to the open field box, the animals underwent OLT and NOR as previously described (see also Supplemental Information) (Antunes and Biala, 2012).

#### Crawley’s Three-Chamber Test

Sociability and social memory were assessed as previously described (Nadler et al., 2004). Each mouse was placed in the apparatus for 2 × 10 min sessions in order to test social interaction and social memory. The interaction was scored manually and a discrimination index was calculated as described above.

#### Rotarod Test

Evaluation of fine motor coordination and balance was assessed on a rotarod apparatus (Ugo Basile, Comerio, Italy) over 5 consecutive days. On the first day, mice underwent one training session at constant speed, consisting of three trials of 120 s each. During the subsequent 4 test days, the rotating rod was set to accelerate from 4 to 40 revolutions per minute over 300 s. Mice were allowed 15 min rest between the trials. Latency to fall was registered once the mouse landed on a lever.

#### T-Maze

The delayed non-matched to position task was carried out as previously described (Deacon and Rawlins, 2006). Prior to and during testing, mice were single caged, food restricted, and habituated to a food reward (1 mL of condensed milk and water in a 1:1 ratio, 1 hr before the dark phase). Mice were weighed daily and kept to 85%–90% of their original weight. Two 10 min habituation trials to the apparatus over 2 days were followed by training/test for 8 days. Each day, every mouse underwent ten trials, each consisting of a forced and a free choice run with 30 s ITIs in the home cage. Right and left goal arms were baited in a random and balanced fashion with 70 μL of milk/water.

### Experimental Design and Statistics

Cell counts, *in vivo* recordings, and behavioral experiments were performed by an investigator blind to the genotypes. Statistical analysis was carried out using Prism 6 version 6.05 for Windows (GraphPad Software, La Jolla, CA, USA). All data were tested for normality using a Kolmogorov-Smirnov test and subsequently analyzed using an appropriate statistical test: unpaired t test, one sample t test against a theoretical mean, and two-way RM ANOVA with post hoc uncorrected Fisher’s LSD, for normally distributed data, and the non-parametric Mann Whitney test for non-normally distributed data. All t tests were two-tailed with an alpha of 0.05. In addition, data from the T-maze experiment were examined via Pearson (r) correlations.

## Author Contributions

Conceptualization, L.M., C.B., C.S.-H., and N.K.; Methodology, L.M., C.B., C.S.-H., and N.K.; Investigation, L.M., C.B., C.S.-H., and P.T.; Writing – Original Draft, L.M. and N.K.; Writing – Review and Editing, L.M., C.B., C.S.-H., P.T., M.H., and N.K.; Funding Acquisition, N.K., C.B., and M.H.

## Supplemental Information

Document S1. Supplemental Experimental Procedures, Figures S1–S4, and Tables S1 and S2

Document S2. Article plus Supplemental Information
