# Supplementary material for: NKX2-1 Is Required in the Embryonic Septum for Cholinergic System Development, Learning, and Memory
Source: Cell Rep. 2017 Aug 15;20(7):1572–84. doi: 10.1016/j.celrep.2017.07.053 (PMC5565637; doi:10.1016/j.celrep.2017.07.053)
Supplement: Document S1. Supplemental Experimental Procedures, Figures S1–S4, and Tables S1 and S2 [file mmc1.pdf]

Cell Reports, Volume *20*

Supplemental Information

NKX2-1 Is Required in the Embryonic Septum for Cholinergic System Development, Learning, and Memory

Lorenza Magno, Caswell Barry, Christoph Schmidt-Hieber, Polyvios Theodotou, Michael Häusser, and Nicoletta Kessaris

Supplemental Information

Supplemental Figures

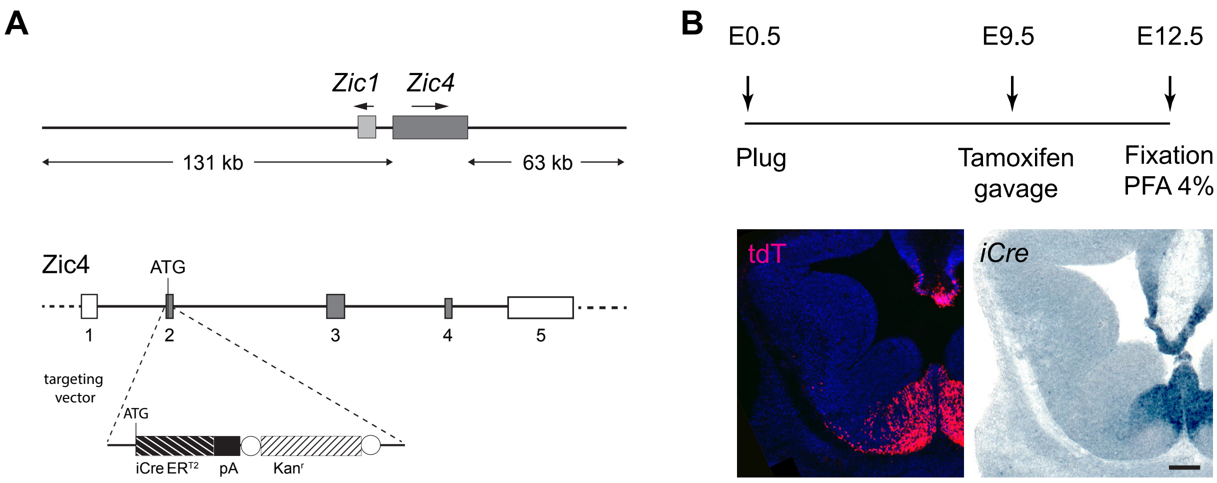

Figure S1, related to Figure 1: Generation of an inducible Zic4-CreER^{T2} transgenic mouse line

(**A**) Strategy for modification of the *Zic4* genomic BAC. Upper row, the unmodified BAC containing *Zic4*. Lower row, strategy for modification of the genomic BAC by insertion of iCreER^{T2}-polyA into the *Zic4* gene. A bacterial kanamycin resistance cassette (Kan^{r}) was included in the targeting vector. This was flanked by FRT sites (open circles) and was removed before microinjection of the modified BAC into fertilized eggs.

(**B**) Assessment of recombination. Upper row, tamoxifen induction protocol. Lower row, detection of tdTomato at E12.5 following induction at E9.5 and *in situ* hybridization for *iCre*. Tdtomato expression recapitulates *iCre* distribution, confirming faithful expression of the transgene.

Scale bar: B: 100 μm

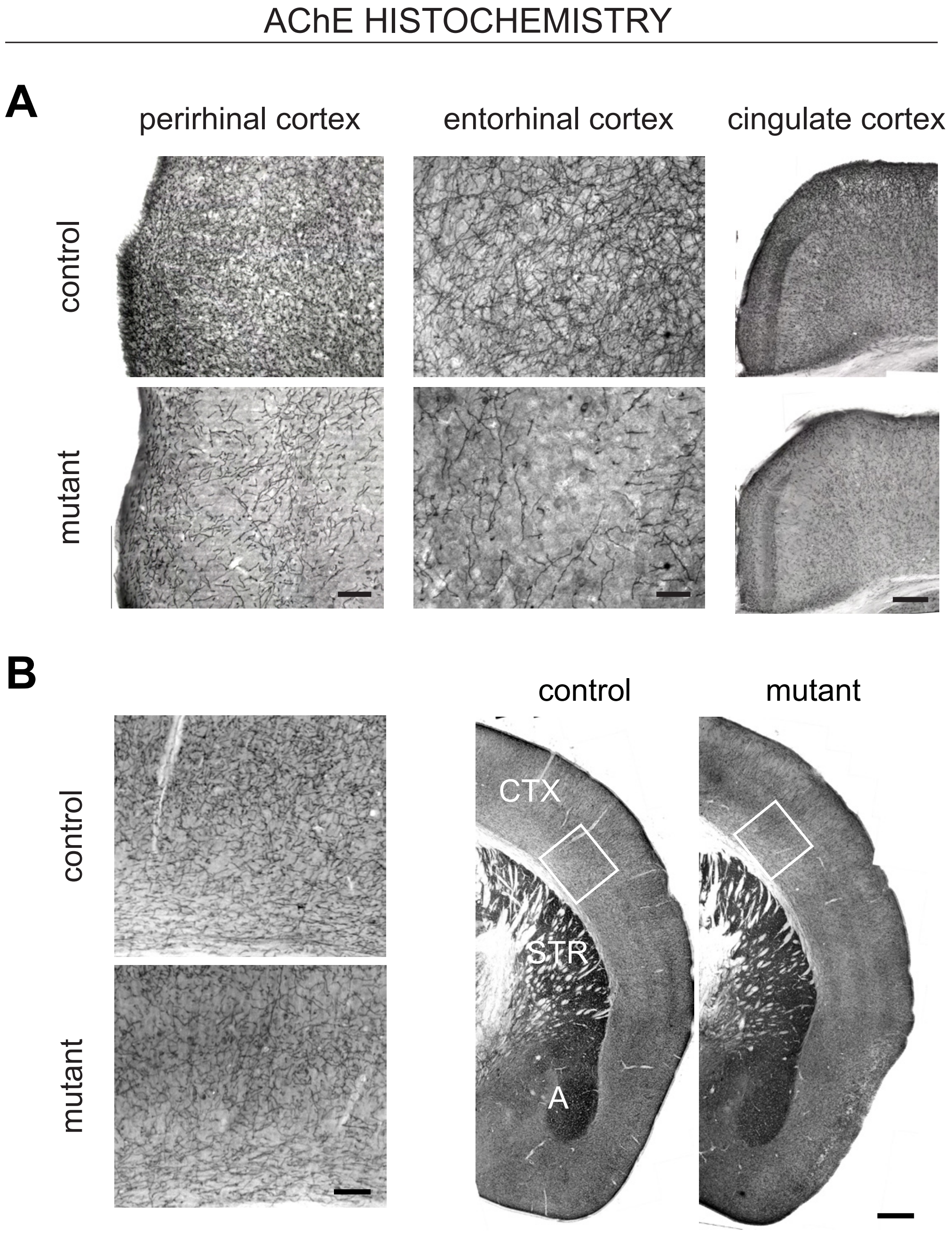

Figure S2, related to Figure 4: Septal ablation of NKX2-1 leads to cholinergic denervation of adult parahippocampal structures

Acetylcholine esterase histochemistry at P90.

(**A**) Loss of cholinergic fibres in the temporal lobe and cingulate cortex of mutant mice compared to controls.

(**B**) Preserved cholinergic innervation in the neocortex, amygdala and caudal striatum in mutant mice compared to controls.

Scale bars: A: perirhinal cortex 100 μm, enthorinal cortex 50 μm, cingulate cortex 200 μm, B: left 100 μm, right 400 μm. CTX, cortex; STR, striatum; A, amygdala.

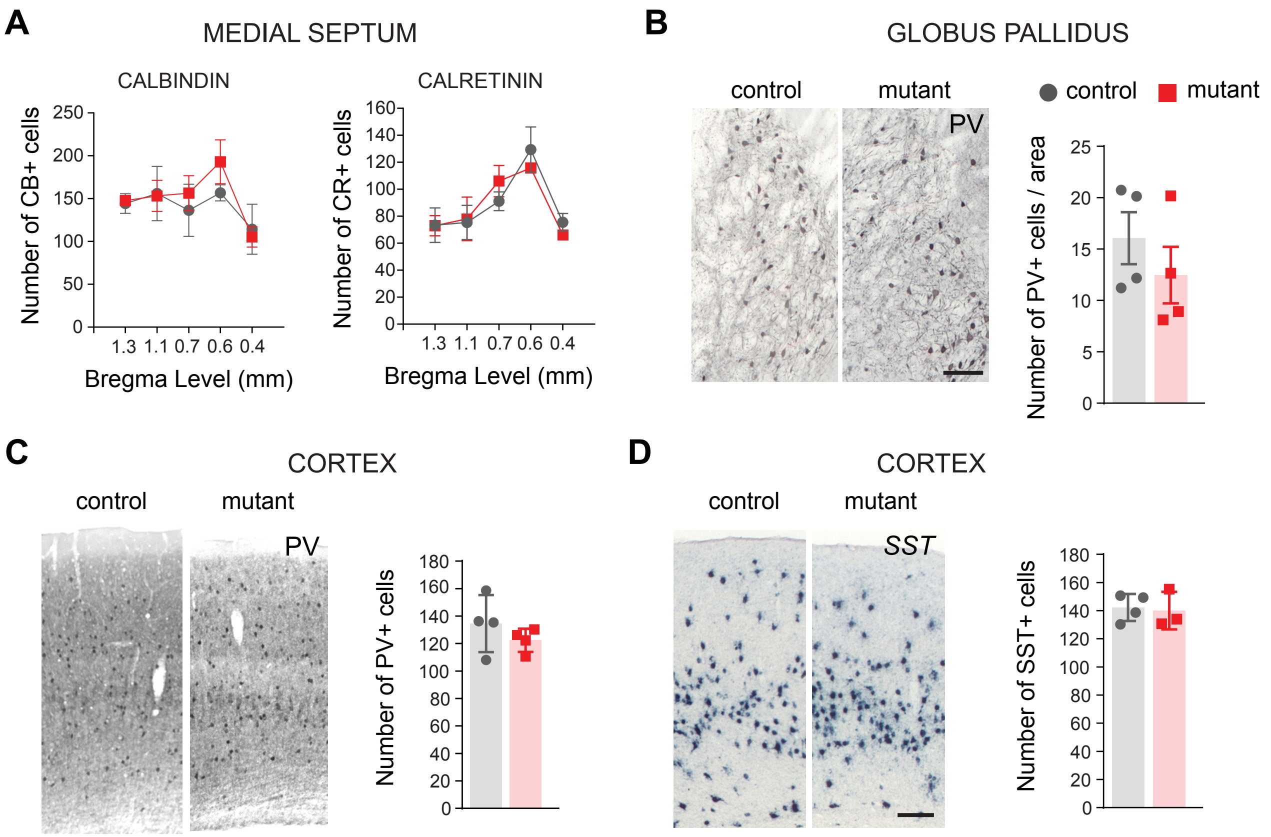

Figure S3, related to Figures 4 and 5: Normal development of septal calretinin and calbindin-expressing neurons, globus pallidus projection neurons and cortical interneurons

(**A**) Normal numbers of CB and CR-expressing neurons in the MSvDB area in mutant mice lacking NKX2-1 in the septum at P30 (mean ± SEM). CB: controls n = 4, mutants n = 5; total numbers: controls, 707. 5± 105.44; mutants, 755.3 ± 73.14; unpaired t-test with Welch's correction p = 0.724. Two-way RM ANOVA: genotype, F(1,6) = 0.1384, p = 0.7226; level, F(4,24) = 6.876, p = 0.0008; interaction, F(4,24) = 1.050, p = 0.4025. CR: controls n = 4, mutants n = 5; total numbers: controls, 439 ± 29.35; mutants, 445 ± 35.87; unpaired t-test with Welch's correction p = 0.886. Two-way RM ANOVA: genotype, F(1,7) = 0.0217, p = 0.8871; level, F(4,28) = 8.41, p = 0.0001; interaction, F(4,28) = 0.532, p = 0.7132.

(**B**) Normal specification of globus pallidus neurons and MGE-derived cortical interneurons. Mutant and control mice show similar numbers of PV-expressing GABAergic neurons. N = 4 mice for each genotype, mean ± SEM: controls, 16.07 ± 2.53 area normalised; mutants, 12.47 ± 2.75 area normalised; unpaired t-test with Welch's correction p = 0.374.

(**C-D**) Cortical GABAergic interneuron numbers are normal in conditional mutant mice. N = 4 per genotype; mean ± SEM. PV: controls, 134.6 ± 10.37; mutants, 122.4 ± 4.242; unpaired t-test with Welch's correction p = 0.339. SST: controls, 142.3 ± 4.79; mutants, 140 ± 7.72; unpaired t-test with Welch's correction p = 0.374.

Scale bars: B: 50 μm; D: 50 μm

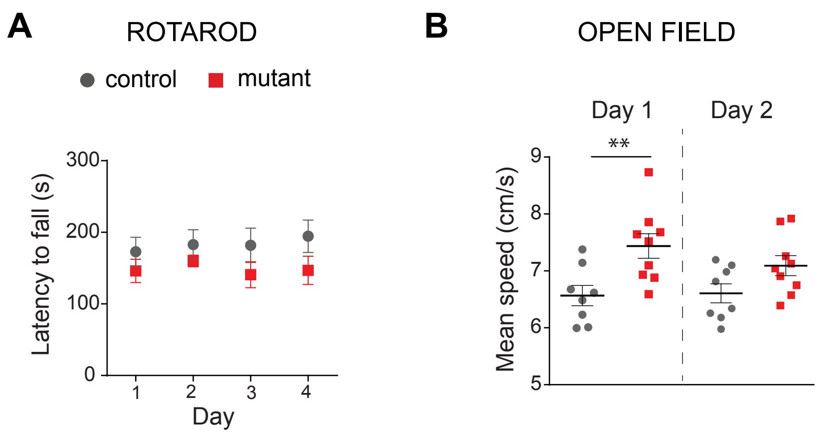

Figure S4, related to Figure 6: Normal motor coordination and locomotor behaviour upon septal NKX2-1 deletion

(**A**) Rotarod test over 4 days, n = 8 per genotype. The latency to fall averaged for three trials each day is shown (± SEM). Mutant mice show normal motor coordination compared to controls. Two-way RM ANOVA: genotype, $F(1, 14) = 2.590$, $p = 0.1298$; day, $F(3, 42) = 0.4127$, $p = 0.7447$; interaction, $F(3, 42) = 0.3616$, $p = 0.7811$.

(**B**) Open field test, controls n = 8, mutants n = 9. Mean speed over a 30 min trial on Day 1, and a 10 min trial on Day 2 (± SEM). Day 1, unpaired t-test with Welch's correction $p = 0.007$; Day 2, unpaired t-test with Welch's correction $p = 0.063$.

Table S1 related to Figure 6: Analysis of total exploration levels during the familiarization, first test session (10 min ITI), second test session (24h ITI) of the NOR and OLT tests

		control	mutant	t test p value
NOR	fam	56.7 ± 5.73 s	66.18 ± 6.66 s	0.301
	10 min ITI	62.15 ± 12.65 s	66.08 ± 7.38 s	0.793
	24h ITI	58.05 ± 4.91 s	37.81 ± 5.05 s	0.011
OLT	fam	28.67 ± 7.03 s	45.07 ± 9.32 s	0.18
	10 min ITI	51.13 ± 6.80 s	55.74 ± 6.80 s	0.638
	24h ITI	43.11 ± 5.78 s	41.64 ± 6.49 s	0.868

Table S2 related to Figure 6: Analysis of spontaneous preference for an object/place during the familiarization phase of the NOR and OLT tests

		control	mutant
NOR	object1	27.79 ± 1.98 s	33.57 ± 4.02 s
	object2	28.95 ± 3.96 s	32.62 ± 3.02 s
	t test p value	0.78	0.853
OLT	left side	11.73 ± 2.57 s	18.05 ± 3.45 s
	right side	12.33 ± 3.73 s	21.62 ± 5.53 s
	t test p value	0.89	0.594

Supplemental Experimental Procedures

Image acquisition and processing

Images were captured on a Zeiss fluorescent microscope with 20x or 10x objectives and assembled into composites using Microsoft ICE software (Microsoft Corp., Redmond, WA). Composites were further processed with Adobe Photoshop CS5 (Adobe Systems Inc., San Jose, CA) for general contrast and brightness enhancements or for conversion into black and white images. The final compositing of the figures was performed with Adobe Illustrator CS5 (Adobe Systems Inc., San Jose, CA).

Cell counts

Quantification of immunolabelled profiles for CHAT and other septal markers was performed at various rostro-caudal levels as outlined in the results section. Counts for PV-immunoreactive cells in the globus pallidus were expressed as number of cells over the area (quantified using Adobe Photoshop). Counts were performed on four representative sections through the rostrocaudal extent of the nucleus for each mouse. Quantification of PV and SST in the somatosensory cortex were carried out as previously described[1] in a defined area spanning the pial–white matter extent of the cortex (1000 μm width and 30 μm depth).

Power spectra analysis

To avoid potential confounds with other behavioural states (e.g. rearing) analyses of the power spectra and calculation of the theta-index were limited to periods when the animals were in motion (speed >= 1 cm/s). Power spectra were calculated by finding the fast Fourier transform of the concatenated filtered data, where the square-modulus of each Fourier frequency coefficient represents the signal power at that frequency. The power spectrum was smoothed using a Gaussian kernel with standard deviation 0.5 Hz. Results were robust to variations in kernel size and shape.

In order to characterise the relationship between theta frequency and running speed we used an additional approach to estimate instantaneous LFP frequencies. The recorded LFP signal was filtered using a 6-12 Hz, 251-tap, Blackman windowed, band-pass sine cardinal filter. An analytic signal was then constructed using the Hilbert transform and takes the form $s_a(t_k) = s(t_k) + i\mathrm{H}[s(t_k)]$, where H specifies the Hilbert transform, $s(t_k)$ is the filtered LFP signal, $t_k = k\Delta$, where $k = 1,\ldots,K$ indexes the time-step and Δ is the inverse of the sampling rate. The phase of the analytic signal $\varphi(t_k)$ gives the phase of the LFP at t_k and the difference in phase between each time point defines the frequency. Thus, concurrent measurements of speed and LFP frequency were produced at theta frequency timescales (around 50 ms). To quantify the relationship between the two variables a regression line was fit to the data from each trial; in order to exclude non-perambulatory activities only results obtained for running speeds between 1 cm/s and 21 cm/s in 4 cm/s bins were considered. In this way we determine the intercept of the regression line with the y-axis, and the slope, defined as the gradient of the regression line.

Behaviour

Novel Object Recognition task

A pilot experiment was run to determine that mice did not prefer an object pair over the others. Mice were given one habituation session to explore the empty arena for 5 min, followed by a 10 min familiarization phase consisting of exposure to two identical objects placed in a symmetrical configuration, and a 10 min test phase, with 10 min or 24 h inter-trial interval. In the test phase, one of the two identical objects was switched with a new object. The time spent interacting with the objects, defined as the physical engagement with the object, or an exploratory activity where the

nose is pointing towards the object and it is no more than 2 cm away from it, was manually scored by two independent observers for the first 5 min of the task, when the novel effect is maximal. A discrimination index (DI) was calculated as the difference between the time spent interacting with the new object and the time spent with the familiar one, over the sum of the time spent interacting with the two objects[2].

Object Location Task

The object location task was carried out as the NOR task with a modification: during the test session mice were exposed to the two equally familiar objects, with one displaced object with respect to the familiarization phase. The position of the displaced object was counterbalanced between mice. A discrimination index (DI) was calculated as the difference between the time spent interacting with the displaced object and the time spent with the familiar one, over the sum of the time spent interacting with the two objects.

Supplemental References

1. Magno,L., Oliveira,M.G., Mucha,M., Rubin,A.N., & Kessaris,N. Multiple embryonic origins of nitric oxide synthase-expressing GABAergic neurons of the neocortex. Front Neural Circuits. 6, 65 (2012).

2. Antunes,M. & Biala,G. The novel object recognition memory: neurobiology, test procedure, and its modifications. Cogn Process 13, 93-110 (2012).
